# Supplementary material for: A cross-country and country-specific modelling of stock market performance, bank development and global equity index in emerging market economies: A case of BRICS countries
Source: PLoS One. 2020 Nov 10;15(11):e0240482. doi: 10.1371/journal.pone.0240482 (PMC7654802; doi:10.1371/journal.pone.0240482)
Supplement: Attachment — Submitted filename: comments.docx [file pone.0240482.s002.docx]

It is of great practical significance to study the interactive relationship between stock markets and banks in BRICS countries.

But I have some comments:

(1) There is a deviation between the development of China's stock market and the development of banks. In other words, banks are developing rapidly, but the stock market is not performing well. The internal mechanism lies in the state's control of banks, which leads to inefficient banks. However, due to the low quality of listed companies and the imperfect accounting system, especially the fraud of financial statements, the stock market has seriously affected the healthy development of the Chinese stock market. Therefore, this article should strengthen the analysis of the internal mechanism.

It is recommended to read the following paper.

Fang, J., Lau, C. K. M., Lu, Z., Tan, Y., & Zhang, H. (2019). Bank performance in China: A Perspective from Bank efficiency, risk-taking and market competition. *Pacific-Basin Finance Journal*, *56*, 290-309.

(2) “Credit to the Private Sector as a ratio of Gross Domestic Product (CPSGDP) is used as a proxy for bank development.” I think this indicator is very inappropriate to measure the development of Bank of China. Because it is not easy for China's private sector to obtain loans and its proportion is relatively small, this will lead to doubts about the reliability of the results.

(3) The development of the bank and the stock market is not only highly related to economic development, but also has some deviations. But the lack of consideration of economic development indicators in the article is a major flaw.

Henry, P. B. (2000). Stock market liberalization, economic reform, and emerging market equity prices. The Journal of Finance, 55(2), 529-564.

Baele, L., De Jonghe, O., & Vander Vennet, R. (2007). Does the stock market value bank diversification?. *Journal of Banking & Finance*, *31*(7), 1999-2023.
