# Supplementary material for: A cross-country and country-specific modelling of stock market performance, bank development and global equity index in emerging market economies: A case of BRICS countries
Source: PLoS One. 2020 Nov 10;15(11):e0240482. doi: 10.1371/journal.pone.0240482 (PMC7654802; doi:10.1371/journal.pone.0240482)
Supplement: Attachment — Submitted filename: Response to Reviewers Comments.docx [file pone.0240482.s003.docx]

Reviewer 1:

Comment #1

There is a deviation between the development of China's stock market and the development of banks. In other words, banks are developing rapidly, but the stock market is not performing well. The internal mechanism lies in the state's control of banks, which leads to inefficient banks. However, due to the low quality of listed companies and the imperfect accounting system, especially the fraud of financial statements, the stock market has seriously affected the healthy development of the Chinese stock market. Therefore, this article should strengthen the analysis of the internal mechanism.

It is recommended to read the following paper.

Response to Comment #1

We thank the reviewer for the above comment which we consider as a very helpful insight into an area of the stock market and bank development nexus in China that we overlooked. Useful additions have been made to the study by not just taking into account the comment but also reading the recommended papers out of which some additions were made to our study, see pages 9 and 21.

Comment #2

“Credit to the Private Sector as a ratio of Gross Domestic Product (CPSGDP) is used as a proxy for bank development.” I think this indicator is very inappropriate to measure the development of Bank of China. Because it is not easy for China's private sector to obtain loans and its proportion is relatively small, this will lead to doubts about the reliability of the results.

Response to Comment #2

We followed prior authors using CPSGDP as a proxy for bank development. These include Rajan and Zingales in *American Economic Review* (1996) Levine in *Journal of Economic Literature* (1997), even more recent studies like Gaies in *Energy Policy* (2019), Maruta in *Journal of Macroeconomics* (2019), including three policy/working papers by the IMF on: (i) *Remittances, Financial Development and Growth* by Paola Giuliano and Marta Ruiz-Arranz (2006); (ii) Financial Development and Economic Growth by Jose De Gregorio and Pablo E. Guidotti (1995) and, iii. Introducing a New Broad-based Index of Financial Development by Katsiaryna Svirydzenka (2016). Undoubtedly, we appreciate the help of the reviewer in pointing out this nuance of the Chinese economy that is of great interest and we noted it on page 10. Suffice it to say that this point should be kept in mind by the readers. The use of CPSGDP here is for comparative and cross-country study purposes. Future studies that are specifically focused on the Chinese economy would represent a good enough platform for exploring this line of measurement for bank development.

Comment #3

The development of the bank and the stock market is not only highly related to economic development, but also has some deviations. But the lack of consideration of economic development indicators in the article is a major flaw.

Response to Comment #3

There is undoubtedly a relationship among bank, stock market and economic development as pointed out by the reviewer. We are thankful for this keen observation. We see financial development as a part of economic development which is of interest to this study, hence we explored bank development and stock market development as disaggregated components of financial development. It is also notable that financial development is a subset of economic development. Moreover, in measuring bank development and stock market development, we scaled credit to the private sector and market capitalization by GDP which is more or less a correlate of economic development. Nevertheless, it needs to be kept in mind that since a unique linear combination of the used variables produced cointegration, it is plausible that an excluded nonstationary variable such as economic development has little or no long run effect.

In addition, the used ARDL approach minimizes problems associated with omitted variables.

**Reviewer 2:**

Comment #1.

The research question is interesting, but the writing is sloppy and unintelligible, and contains many errors. I believe that an improvement on the writing by an editorial expert will significantly increase the quality of the paper.

Response to Comment #1.

We gratefully acknowledge the constructive and helpful comments provided by this referee. We believe that sufficient care has been taken to address the referee’s comments. Most importantly, the paper has greater clarity because of the reviewer’s helpful comments. For this, the authors wish to thank the referee.

Comment #2.

The introductory section guides readers to understand the motivation, the importance and the contribution of the paper through a winding road of one thousand miles without a clear route.

Response to Comment #2.

The paper also benefited from fruitful comments concerning the introduction. We have retouched it to make it more pointed and better directed. We are very grateful to the referee.

Comment #3.

The section of literature review looks more like a summary on papers cited. This section should systematically analyze, properly synthesize and critically evaluate the literature to deliver a clear picture of the state of knowledge on the subject, and figure out the gaps and to which you would like to contribute.

Response to Comment #3.

The literature section has been rewritten and it now looks better than the original submission, thanks to the reviewers.

Comment #4.

4. I fail to find out what LCPSGDP shown in Eq. (1) and other equations represents. Should it be CPSGDP?

Response to Comment #4.

The referee is correct and we have fixed that by correcting the interchangeable use of CPSGDP in its level and log forms has been corrected and a properly harmonized presentation made in the revised manuscript.

Comment #5.

As the authors said, the developments of the banking and the stock market could affect each other, so that a model that can address the reverse causation is essential. However, the authors did not do so, even though they present two equations of each of the two developments as the dependent variable in their paper.

The authors simply run regressions for each of the two equations, which is not the right way to address the reverse causation (endogeneity). The two equations should be simultaneously estimated in a system. Some proper techniques such as VAR model and simultaneous equation model can be used.

Response to Comment #5.

Following the referee’s comments, we have tested endogeneity using the VAR method and the result is in table 5 page 20.

General Comment

The comments by the Editor were also addressed in the revised manuscript.

This study as revised is obviously better than its original form, thanks to the reviewers for their useful and very valid comments. The authors unreservedly express their gratitude to them for this good work and most of all to the editor for his uncommon ability in driving the paper through the publication process.
