# Supplementary material for: A cross-country and country-specific modelling of stock market performance, bank development and global equity index in emerging market economies: A case of BRICS countries
Source: PLoS One. 2020 Nov 10;15(11):e0240482. doi: 10.1371/journal.pone.0240482 (PMC7654802; doi:10.1371/journal.pone.0240482)
Supplement: Attachment — Submitted filename: response to reviewer 3.docx [file pone.0240482.s004.docx]

Reviewer #3:

**Introduction**

We most sincerely apologise to reviewer #3 for the inadvertent omission of our response to his excellent sets of comments. Permit me to say that it was not deliberate but an innocent omission. Reviewer 3 is among the best in terms of detailed comments and helpful review of our study and does not in any way deserve the regrettable omission of our response to the helpful comments. We have responded as requested to the 8 comments raised by the reviewer and undoubtedly the paper is better than the original and revised (R1) submission; thanks to the help of Reviewer 3.

Comment #1.

The whole paper needs to be proofread to get rid of typos and grammar issues.

Response to Comment #1.
As suggested by the referee, we employed the services of a professional editor who proofread the entire paper at a significant cost. We are grateful to the referee for this suggestion.

Comment #2.

Starting from the introduction, the authors argue that "In the light of the above, our enquiry is driven by three key considerations" however, the authors in detail talked about, one... second, third, fourth..... this is a contradiction.

Response to Comment #2.

The observation by the reviewer is quite true and the observed contradiction in numbering the “considerations” has been put in order. We are grateful to the reviewer for his helpfulness.

Comment #3.
It is recommended that the authors should discuss the significance of this study and contributions of the work in a separate and clearer manner. In other words, why this topic is important for BRIC and can the authors provide some detail evidence for each of these four countries to illustrate the importance of this topic and then clearly talked about the gap and their contribution.

Response to Comment #3.

Following the reviewer’s suggestion, page 3 addresses the relevance of this study for the BRICS countries see also table 1 with the highlighted nuances of the BRICS countries and the discussions on how the study will be of significance to the respective BRICS countries. Many thanks to the reviewer for this comment.

Comment #4.

In terms of the literature review, it is recommended that hypothesis development should be also established in this section in accordance to the main aims/objectives of this work. also some of the related works in related to the relationship between stock market development and banking sector development are missing from the literature review, I just listed a few below:

Tan, Y., and Floros, C. (2012a). Bank profitability and inflation: the case of China. Journal of Economic Studies, 39, 675-696.
Tan, Y., and Floros, C. (2012b). Bank profitability and GDP growth in China: a note. Journal of Chinese Economic and Business Studies, 10, 267-273.

Response to Comment #4.

The literature section has entirely been rewritten to capture the stream of argument in the bank and stock market development interactions. More studies including the two suggested by the reviewer have been added to the literature section and has been made more robust than it was originally. In addition, the main thesis of the study has also been captured with the gap that this study intends to fill (see the last two paragraphs in the literature section pg. 9). We also included the three key hypotheses of interest to this study. Thanks to the reviewer for this comment.

Comment #5.

Related to the measurement of variable, the banking sector development is measured by the ratio of credit to the private sector to GDP in the current study, while this is different from Tan (2016) who use the ratio of banking sector assets to GDP, the authors are expected to provide critical discussion on this.

Tan, Y. (2016). The impacts of risk and competition on bank profitability in China. Journal of International Financial Markets, Institutions and Money, 40, 85-110.

Response to Comment #5.

A discussion on this has been provided to justify the use of CPSGDP as a proxy for bank development. We thank the reviewer for making us provide a justification for the parameter used in measuring bank development (See footnote 1, page 10).

Comment #6.

Correlation matrix should be provided to test the issue of multicollinearity.
Response to Comment #6.

In response to the recommendation of reviewer 3, the correlation matrix has been added as table 4 in the study. Given that the variance-covariance matrix can be inverted following our result, multicollinearity appears to have a tolerable effect and has further been contained by transformation of the series (See Berndt, 1996: pp 339) .

Comment #7.
I cannot see any critical discussion about the results through providing linkage and comparison with existing empirical studies.

Response to Comment #7.

The result section has been rewritten to link the findings with our aprior expectation as well as existing empirical studies. Thanks to this reviewer for this excellent comment.

Comment #8.
It is recommended that the authors should talk about three issues in the conclusion in a clearer manner including the summary of the significance, contributions of the study; main results; as well as the policy implications and areas of future research.

Response to Comment #8.

The conclusion segment of this study has been disaggregated to capture the salient issues raised by this reviewer. It is our firm belief that the section has greater clarity and direction, thanks to the insightful comment by the reviewer.
