# Supplementary material for: A cross-country and country-specific modelling of stock market performance, bank development and global equity index in emerging market economies: A case of BRICS countries
Source: PLoS One. 2020 Nov 10;15(11):e0240482. doi: 10.1371/journal.pone.0240482 (PMC7654802; doi:10.1371/journal.pone.0240482)
Supplement: Attachment — Submitted filename: response to reviewer 3.docx [file pone.0240482.s005.docx]

Reviewer #3:
Comment #1.

In terms of the literature review, it is recommended that hypothesis development should be also established in this section in accordance to the main aims/objectives of this work. The hypotheses below were integrated as part of the literature section:

H_o1_: Bank development does not drive stock market development

H_o2_: Stock Market development does drive bank development

H_o3_: Global equity index has no significant impact on bank and stock market development.

The above hypotheses were relocated to the beginning part of the literature section making them provide definite direction for the overall development of the literature review section.

Comment #2.

Correlation matrix should be provided to test the issue of multicollinearity.

Response to Comment #2.

In response to the recommendation of reviewer 3, the correlation matrix has been added as table 4 in the study. Given that the variance-covariance matrix can be inverted following our result, multicollinearity appears to have a tolerable effect and has further been contained by transformation of the series (See Berndt, 1996: pp 339).

Additionally, we created the country specific multicollinearity diagnostics using three measures:

-condition numbers

-Variance Inflation Factors

-Tolerance Ratio

All these measures agree with the earlier established position by the panel correlation matrix which is presented at the lower part of table 4.

Comment #3.

Missing references compared to related in-text citations

Response to Comment #3.

The reference section has been revisited and the missing references have been added. We thank Reviewer 3 for his/her keen observation.
