# Supplementary material for: A cross-country and country-specific modelling of stock market performance, bank development and global equity index in emerging market economies: A case of BRICS countries
Source: PLoS One. 2020 Nov 10;15(11):e0240482. doi: 10.1371/journal.pone.0240482 (PMC7654802; doi:10.1371/journal.pone.0240482)
Supplement: Attachment — Submitted filename: Referee Report on Manuscript PONE-D-20-10606.pdf [file pone.0240482.s001.pdf]

Referee Report on Manuscript PONE-D-20-10606:

A Cross-Country and Country-Specific Modelling of Stock Market Performance, Bank Development and Global Equity Index in Emerging Market Economies: A Case of BRICS Countries

Comments:

1. The research question is interesting, but the writing is sloppy and unintelligible, and contains many errors. I believe that an improvement on the writing by an editorial expert will significantly increase the quality of the paper.

2. The introductory section guides readers to understand the motivation, the importance and the contribution of the paper through a winding road of one thousand miles without a clear route.

3. The section of literature review looks more like a summary on papers cited. This section should systematically analyze, properly synthesize and critically evaluate the literature to deliver a clear picture of the state of knowledge on the subject, and figure out the gaps and to which you would like to contribute.

4. I fail to find out what *LCPSGDP* shown in Eq. (1) and other equations represents. Should it be *CPSGDP*?

5. As the authors said, the developments of the banking and the stock market could affect each other, so that a model that can address the reverse causation is essential. However, the authors did not do so, even though they present two equations of each of the two developments as the dependent variable in their paper.

The authors simply run regressions for each of the two equations, which is not the right way to address the reverse causation (endogeneity). The two equations should be simultaneously estimated in a system. Some proper techniques such as VAR model and simultaneous equation model can be used.
